# Dysregulation of tissue and serum microRNAs in organ transplant recipients with cutaneous squamous cell carcinomas

**DOI:** 10.1002/hsr2.205

**Published:** 2020-11-19

**Authors:** Alexandra Geusau, Liliane Borik‐Heil, Susanna Skalicky, Michael Mildner, Johannes Grillari, Matthias Hackl, Raute Sunder‐Plassmann

**Affiliations:** ^1^ Department of Dermatology Medical University of Vienna Vienna Austria; ^2^ TAmiRNA GmbH Vienna Austria; ^3^ Department of Dermatology, Research Division of Biology and Pathobiology of the Skin Medical University of Vienna Vienna Austria; ^4^ Ludwig Boltzmann Institute for Experimental and Clinical Traumatology Vienna Austria; ^5^ Christian Doppler Laboratory for Biotechnology of Skin Aging, Department of Biotechnology Institute for Molecular Biotechnology, BOKU – University of Natural Resources and Life Sciences Vienna Austria; ^6^ Department of Laboratory Medicine Medical University of Vienna Vienna Austria

**Keywords:** biomarker, immunosuppressed transplant recipients, miRNA, next generation sequencing, skin cancer

## Abstract

Micro RNAs (miRNAs) are considered as promising biomarkers for skin cancer. By next generation sequencing (NGS), we measured and compared miRNA expression profiles of tumor, peri‐lesional, and control tissue of immunosuppressed organ transplant recipients (OTR), suffering from localized cutaneous squamous cell carcinoma (cSCC). Out of 490 miRNAs detected in cSCC tissue, 23 significantly regulated miRNAs were selected for quantitative targeted analysis in serum samples of our patients and control sera from OTR without cSCC. Two onco‐miRNAs (mir‐1290, mir‐1246), previously identified as crucial drivers for tumor initiation and cancer progression, were significantly and exclusively upregulated in both tumor tissue and serum. This finding suggests that miRNA dysregulation in cSCC tissue is reflected in serum even in patients without advanced disease and highly differentiated cSCCs.

## Graphical abstract

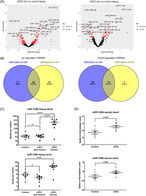

## 1 INTRODUCTION

MicroRNAs (miRNAs) are essential regulators of various cellular processes such as cell growth, differentiation, apoptosis, and the immune response. They are considered to be promising biomarkers and therapeutic targets for different pathological conditions including skin cancer.^1^ MicroRNA profiling may aid in the prediction and diagnosis of cutaneous squamous cell carcinoma (cSCC), and could in the future be used for early detection of subclinical metastases in patients after excision of high‐risk cSCCs. This is particularly important in immunosuppressed organ transplant recipients (OTR), who are at higher risk for keratinocyte cancers (KCs) such as cSCC, since in such patients tumors tend to grow much faster and precancerous lesions rapidly turn into invasive skin tumors.^2^, ^3^ Hence, we tried to identify a miRNA signature suitable for monitoring OTR with cSCC.

## 2 METHODS

### 2.1 Experimental design

We prospectively assessed eight OTR (5 kidney‐, 1 lung‐, 2 heart transplant recipients) who had a cSCC big enough for a 3 mm punch biopsy of lesional as well as perilesional tissue (tumor characterization: Table S1). In addition, a second 3 mm punch biopsy was taken from healthy skin not normally exposed to UV‐light, that is, the gluteal region, and blood was drawn for serum miRNA analysis. Another eight OTR who never before had a skin tumor diagnosed served as control group: each of the eight cases in the study was matched by a control patient of the same gender, who had the same type of organ transplanted at the same age (range 1 year) and was in the same post‐transplantation period (range 1 year). Exclusion criteria were patient age <18 years, malignant condition/cancer other than a cSCC, and any systemic infectious or inflammatory conditions.

We analyzed miRNA expression profiles in the lesional and perilesional tissue samples by next generation sequencing (NGS); a comparison with the samples taken from normal gluteal skin of the same patient served as intraindividual control. Based on these NGS data, a subset of 23 significantly regulated miRNAs was selected for quantitative targeted analysis in serum samples of the cSCC patients and the tumor‐free control group. We analyzed two selected miRNAs for their release into supernatants of cultured primary keratinocytes and three different cSCC cell lines. The supplementary materials offer a detailed description of methods used for total RNA extraction, NGS, and miRNA quantification.

### 2.2 Statistical analysis

Group‐wise differential expression analysis was performed based on spike‐in normalized delta Cq‐values (dCqs) under the assumption of normal distribution, which was assessed visually, using two‐sided *t* tests.

### 2.3 Ethical considerations

The Ethical committee of the Medical University of Vienna approved the study (Number 1233/2016) and all individuals included in the study gave their informed written consent.

## 3 RESULTS

**FIGURE 1:**
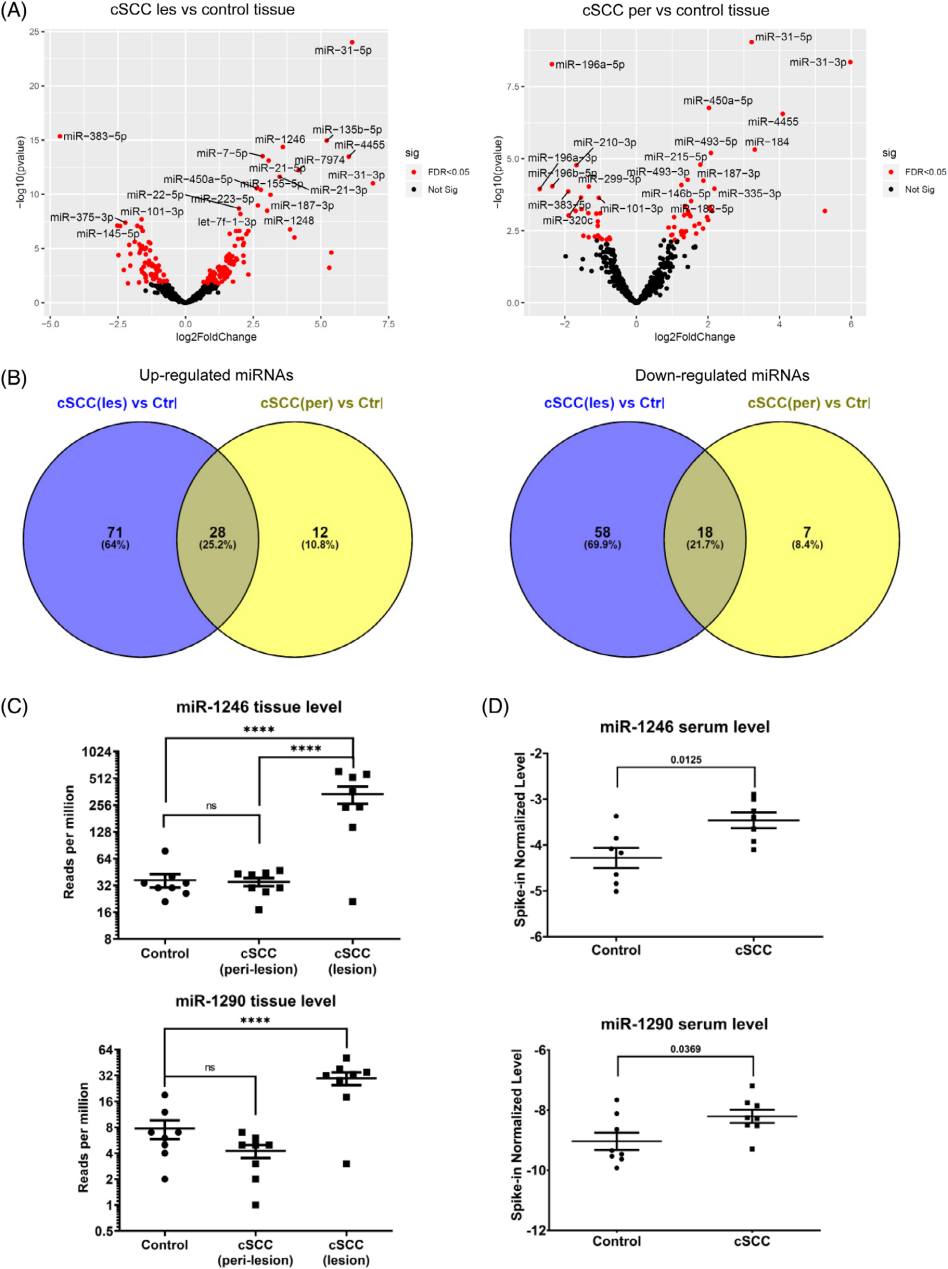
MiRNA expression in tissue and serum of cSCC patients and controls. A, Volcano Plot of 175 miRNAs differentially expressed in lesional vs gluteal tissues (left side) and 65 miRNAs differentially expressed in peri‐lesional vs gluteal tissues (right side); the top 20 miRNAs by *P*‐value (unadjusted) are annotated in the plot. Red color indicates miRNAs with adjusted *P*‐value below .05 (FDR <0.05%). B, Venn overlap of differentially regulated miRNAs: up‐regulated (left side) and down‐regulated (right side). C and D, Scatterplots of miR‐1246 and miR‐1290 up‐regulated in tissue (C) and serum (D). The scatterplots depict miRNA levels in three skin tissue compartments (control, peri‐lesional, and lesional) as well as the serum miRNA levels in tumor patients compared to matched controls. les, lesional; per, peri‐lesional; control (gluteal, UV‐unexposed skin). **** *P* < .05; ns, not significant

Out of 490 miRNAs detected in lesional cSCC tissue (Table S1), 175 were significantly up‐ (n = 99) or downregulated (n = 76) compared to gluteal skin (false discovery rate [FDR] < 0.05; Figure 1A). When comparing miRNA regulation between lesional and perilesional tissue, out of the 99 upregulated miRNAs, 71 were increased in cSCC tissue only (with 27 showing fourfold upregulation; logFC>2), whereas 28 were upregulated in both lesional and perilesional tissue (Figure 1B). Additionally, out of 65 miRNAs significantly regulated (FDR < 0.05) in perilesional vs gluteal skin (Figure 1A; Table S2), 12 miRNAs were increased in perilesional tissue only (Figure 1B). Vice versa, out of the 76 downregulated miRNAs in lesional tissue compared to gluteal (control) skin (Figure 1A), 58 were exclusively downregulated in the tumor (with 10 downregulated >2LogFC) and 7 (out of 25) in perilesional tissue, whereas 18 had a decreased expression in both kinds of tissue (Figure 1B). Thus, in our study, miRNA dysregulation was more pronounced in tumor compared to perilesional tissue, with mostly a stepwise in‐ or decreased expression from control to perilesional to tumor tissue. Interestingly, the miRNAs exclusively upregulated in perilesional tissue were predominantly suppressor‐miRNAs and clearly outweighed those downregulated, being mainly onco‐miRNAs. This inverse regulation might be interpreted as a local response to counter tumor growth.

Based on the NGS data, a panel of 23 miRNAs significantly up‐ or downregulated in lesional (n = 9), perilesional (n = 4), or both (n = 6) tissues, was selected for quantification in serum samples from tumor patients (n = 8) and matched OTRs without cSCC (n = 8).

Mir‐135b, mir‐383, mir‐1248, and mir‐187‐3p levels were below the limit of detection, thus 19 miRNAs remained in the analysis (Table 1). The majority (n = 11) of these 19 miRNAs was downregulated in tumor patients compared to matched controls, for two miRNAs (miR‐27a‐3p, miR‐223‐3p) the difference was significant (*P* < .05). Only two of the 19 miRNAs were significantly and exclusively upregulated in both cSCC and serum: miR‐1290 (*P* = .04) and miR‐1246 (*P* = .01; *P*‐value after exclusion of an outlier using the ROUT test in each group), previously described as onco‐miRs (Table 1, Figure 1C,D).^4^ Additionally, compared to cultured primary keratinocytes, we detected increased expression of both miRNAs in the supernatants of three different cSCC cell lines (Figure S2).

**TABLE 1:** Expression of selected miRNAs in tissue and serum of tumor patients compared to matched controls (n = 19)

		Tissue					Serum		
			cSCC lesional		Peri‐lesional		cSCC vs control		
miRNA ID	S/O°	Regulation in tissue^b^	log2 FC	*P* value^a^	log2 FC	*P* value^a^	Regulation	log2 FC	*P* value^a^
hsa‐miR‐1246	O	Lesional ↑	3.59	**.013**	−0.09	.840	**↑**	**0.44**	**.013**
hsa‐miR‐7‐5p	S	Lesional ↑	2.85	**.000**	0.90	**.011**	↓	−0.82	.183
hsa‐miR‐1290	O	Lesional ↑	2.32	**.000**	−0.85	.054	**↑**	**0.83**	**.037**
hsa‐miR‐223‐3p	S	Lesional ↑	1.73	**.000**	0.39	.349	↓	−1.80	**.019**
hsa‐miR‐424‐5p	O	Lesional ↑	1.65	**.001**	0.67	.119	↓	−0.84	.112
hsa‐miR‐126‐3p	S	Lesional ↑	0.98	**.011**	0.44	.251	↓	−0.65	.124
hsa‐miR‐27a‐3p	O	Lesional ↑	0.91	**.018**	−0.42	.276	↓	−0.97	**.033**
hsa‐miR‐335‐3p	S	Peri‐lesional ↑	0.88	.106	2.18	**.000**	↓	−0.94	.263
hsa‐miR‐378a‐3p	S	Peri‐lesional ↑	0.52	.363	2.06	**.001**	=	−0.19	.665
hsa‐miR‐34a‐5p	S	Peri‐lesional ↑	0.41	.164	0.90	**.002**	=	0.25	.566
hsa‐miR‐148a‐3p	S	Peri‐lesional ↑	0.32	.348	1.06	**.002**	↓	−0.84	.109
hsa‐miR‐31‐5p	O	Lesional + Peri‐lesional ↑	6.15	**.000**	3.22	**.000**	↓	−0.58	.290
hsa‐miR‐21‐5p	O	Lesional + Peri‐lesional ↑	3.07	**.000**	1.36	**.000**	=	−0.25	.508
hsa‐miR‐155‐5p	O	Lesional + Peri‐lesional ↑	2.78	**.000**	1.33	**.001**	↓	−1.19	.096
hsa‐miR‐22‐5p	S	Lesional + Peri‐lesional ↑	1.97	**.000**	1.05	**.001**	↓	−0.71	.068
hsa‐miR‐145‐5p	S	Lesional ↓	−2.52	**.000**	−0.34	.446	=	−0.18	.776
hsa‐miR‐30a‐5p	S	Lesional ↓	−1.11	**.001**	−0.25	.425	=	−0.05	.894
hsa‐miR‐10a‐5p	S	Lesional + Peri‐lesional ↓	−2.08	**.000**	−0.91	**.038**	↓	−0.43	.330
hsa‐miR‐125b‐5p	S	Lesional + Peri‐lesional ↓	−1.58	**.000**	−0.7	**.021**	↑	0.47	.540

Numbers in bold indicate significantly upregulated miRNA in tumor and in serum.

Abbreviations: S, suppressor‐miRNA; O, Onco‐miRNA; log2FC, log2 fold change.

a Significant values (*P* < .05) are in bold.

b Regulation of miRNA expression in cSCC tumor or peri‐lesional tissue compared to healthy gluteal skin.

## 4 DISCUSSION

Many pathways associated with tumor‐growth and ‐progression known to be involved in cSCC development are regulated by miRNAs.^1^ In our study, of the 58 miRNAs exclusively downregulated in cSCC (Table S1), 43 were identified as suppressor‐miRNAs, suggesting an increased expression of their oncogenic target proteins in KCs. This scenario may be exemplified with miR‐145‐5p, which showed a reduction in tumor tissue of −2.52 log_2_FC (corresponding to a reduction by 82.5%; Table 1). Recently, Gao et al showed that miRNA is involved in regulating apoptosis, stem cell features, and the metabolism of laryngeal squamous cell carcinoma cells.^5^ We found that the expression of miR‐145 in the serum of our cSCC patients was not significantly altered. A possible explanation could be the systemic expression of this particular miRNA across various tissues, specifically in smooth muscle cells.^6^

In contrast, two primate‐specific onco‐miRNAs (miR‐1290, miR‐1246) were significantly and exclusively upregulated in both cSCC and serum. Additionally, compared to cultured primary keratinocytes, we observed increased expression of both miRNAs in the supernatants of three different cSCC cell lines (Figure S2). Target genes of both miRNAs (1246:290 targets, 1290:148 targets according to Tarbase^7^) are enriched in various biological pathways including apoptosis or p53 signaling. Sun et al recently reported that tissue miR‐1290 levels were highly correlated with serum miR‐1290 levels in esophageal SCC, and high serum levels were significantly associated with poor survival.^8^ In a tumor‐initiating cell model, miR‐1246 and miR‐1290 were identified as crucial drivers for tumor initiation and progression in human non‐small cell lung cancer.^4^ Sun et al speculated that direct inhibition of either miRNA could arrest the growth of established patient‐derived xenograft tumors, thus indicating that these miRNAs are clinically useful as biomarkers for tracking disease progression and as therapeutic targets.

In our study, the number of patients and the number of miRNAs quantified in serum samples was small and the selection for serum investigation was based on miRNA expression patterns in tumor tissue. For the first time we are able to show that miRNAs dysregulated in lesional and perilesional cSCC tissue (compared to healthy skin of OTR) are also found to be dysregulated in the patient serum. The upregulation of the onco‐miRNAs miR‐1246 and miR‐1290 in serum of OTRs suffering from cSCC is remarkable. It suggests that miRNA dysregulation in cSCC tissue might be reflected in serum even in patients without advanced disease and highly differentiated cSCCs (Table S1). Thus, quantification of these miRNAs in serum might give an insight into the activity of the main tumor and may serve as a tool for the identification of cSCC recurrence and metastases. In theory, miRNA profiling could aid in the prediction of outcomes and the design of new, targeted therapies. Finally, we would hope that an extended miRNA expression analysis in serum samples from our patient collective, with confirming data from a larger cohort, might reveal a serum miRNA signature specific to cSCC.

## CONFLICT OF INTEREST

Matthias Hackl and Johannes Grillari are co‐founders and shareholders of TAmiRNA GmbH. All other authors state no conflict of interest.

### TRANSPARENCY STATEMENT

Alexandra Geusau affirms that this manuscript is an honest, accurate, and transparent account of the study being reported ‐ and that no important aspects of the study have been omitted.

### AUTHOR CONTRIBUTIONS

Conceptualization: Alexandra Geusau, Raute Sunder‐Plassmann

Data Curation: Liliane Borik‐Heil Formal Analysis: Matthias Hackl, Susanna Skalicky, Michael Mildner Funding Acquisition: Alexandra Geusau

Investigation: Susanna Skalicky, Michael Mildner, Liliane Borik‐Heil Project Administration: Alexandra Geusau, Raute Sunder‐Plassmann

Resources: Alexandra Geusau, Liliane Borik‐Heil, Michael Mildner

Visualization: Matthias Hackl, Liliane Borik‐Heil, Michael Mildner, Raute Sunder‐Plassmann, Alexandra Geusau Writing – Original Draft Preparation: Alexandra Geusau, Raute Sunder‐Plassmann

Writing – Review & Editing: Matthias Hackl, Alexandra Geusau, Raute Sunder‐Plassmann, Johannes Grillari

 All authors have read and approved the final version of the manuscript.

 Alexandra Geusau had full access to all of the data in this study and takes complete responsibility for the integrity of the data and the accuracy of the data analysis.

## Supporting information


**Appendix S1**. Supporting Information

**Table S1**

**Table S2**

## Data Availability

The authors affirm that the data supporting the findings of this study are available within the article and/or its supplementary materials.
