# Supplementary material for: Dysregulation of tissue and serum microRNAs in organ transplant recipients with cutaneous squamous cell carcinomas
Source: Health Sci Rep. 2020 Nov 19;3(4):e205. doi: 10.1002/hsr2.205 (PMC7676459; doi:10.1002/hsr2.205)
Supplement: Supplementary file 1 — Appendix S1. Supporting Information [file HSR2-3-e205-s001.docx]

**Supplemental material**

**Patients and Methods**

**1. Study population:** Organ transplant recipients (OTR) from the Dermatological Department of the Medical University of Vienna. The Study was approved by the Ethical Committee of the Medical University of Vienna (Number **1233/2016**), and all patients included in the study gave their informed written consent.

- OTR Cases: 8 OTR (5 kidney-, 1 lung-, 2 heart transplant recipients) who had a biopsy-verified cutaneous squamous cell carcinoma (cSCC), prior to excision. The size/diameter of the tumor had to be at least 1 cm^2^ to ensure a meaningful histological investigation.

- OTR Controls: 8 OTR who never before had a skin tumor diagnosed. Each of the eight cases in the study was matched by a control patient of the same gender, who had the same type of organ transplanted at the same age (range one year), and was in the same post-transplantation period (range one year.

Exclusion criteria were: age <18 years, any malignant condition or cancer other than cSCC, systemic infections or any other inflammatory conditions; diameter of cSCC below 1cm^2^.

Patient characterisation included very detailed patient interviews, a thorough dermatological investigation, the objective determination of skin pigmentation (Fitzpatrick score, photometric analysis of skin colour), assessment of clinical signs of skin aging, and the estimation of the total Ultraviolet (UV) burden (based on patient interviews and latitude and altitude of reported outdoor activities over time).

**Table S1:** Tumor characterisation and stage of eight OTR

**2. Methods:**

**2.1 Patient related measures:**

Newly diagnosed skin tumours were first biopsied and later excised. From the excisional material of at least 1 cm^2^ size, two 3 mm punch biopsies were taken from i) lesional tissue and ii) perilesional skin. Additionally, a 3 mm punch biopsy was obtained from normal UV-unexposed skin (gluteal) from the cases. Blood for serum miRNA analysis was drawn from each case and control subject.

**2.2 miRNA analysis** of tissue, serum and conditioned media samples

2.2.1 Tissue-based microRNA analysis

- Total RNA extraction from skin biopsies

Skin tissue biopsies were homogenised on dry ice using TissueRuptor disposable knives (Qiagen, Germany). Total RNA was extracted from homogenised (Qiazol, Qiagen, Germany) tissues using phenol/chloroform extraction, precipitation and elution using the miRNeasy Mini purification kit (Qiagen, Germany). Total RNA was eluted from purification columns in 30 µl nuclease-free water. RNA integrity was checked using the RNA 6000 Nano Assay (Agilent, CA) as well as spectrophotometric RNA quantification (Nanodrop).

- Next-generation sequencing

Defined amounts of total RNA (between 10 and 100 ng) were used for small RNA library preparation using the NEBNext library preparation kit (New England Biolabs, US). Adapter-ligated libraries were amplified (17 cycles) using barcoded Illumina reverse primers in combination with the Illumina forward primer. A pool containing equimolar amounts of the 24 independently generated small RNA sequencing libraries was prepared on the basis of concentrations derived from DNA-1000 high-sensitivity bioanalyaser analysis (Agilent, CA). Sequencing was performed on an Illumina HiSeq 2500 with 50 bp single-end reads. Sequencing reads were adapter trimmed and filtered for low-quality reads (Q < 30). MicroRNA annotation was performed on the basis of sequence alignments against the genome reference and miRBase release 22 using bowtie 2.0 taking advantage of the tools available under the sRNA toolbox (<https://www.ncbi.nlm.nih.gov/pmc/articles/PMC4489306/>). Read counts were normalised to the total number of reads detected per sample to obtain the “tags per million” (TPM) for each miRNA and sample.

- Data Analysis

Exploratory data analysis was performed using normalised read counts and the publicly available tools ClustVis (Metsalu, Tauno and Vilo, Jaak. Clustvis: a web tool for visualizing clustering of multivariate data using Principal Component Analysis and heatmap. Nucleic Acids Research, 43(W1):W566–W570, 2015. doi: 10.1093/nar/gkv468). Principal component analysis (PCA) and hierarchical clustering analyses were performed using the default settings, i.e. singular value decomposition with imputation, Pearson correlation as the distance metric, and average distance for clustering. P-values for effects on microRNA transcription (log2 transformed fold changes) were estimated using EdgeR (Robinson MD, McCarthy DJ, Smyth GK (2010). “edgeR: a Bioconductor package for differential expression analysis of digital gene expression data.” Bioinformatics, **26**(1), 139-140) under R/Bioconductor, and adjusted for multiple testing based on the method of Benjamini-Hochberg (Controlling the False Discovery Rate: A Practical and Powerful Approach to Multiple Testing, <http://www.jstor.org/stable/2346101>, doi 10.2307/2346101) (**Figure S1**).

2.2.2 microRNA analysis in serum and conditioned media

- Total RNA extraction

Conditioned media was obtained from KC cultures through centrifugation at 500xg for 15 min (pellet discarded), followed by centrifugation at 14,000xg for 15 min (pellet discarded), and finally 0.8µm filtration. The volume of conditioned media was reduced by ultrafiltration using Amicon filters with 10 kDa size cut-off, retaining both protein complexes and extracellular vesicles in solution.

Total RNA was extracted from 200 µl serum or 200 µl concentrated conditioned media (CCM) using the miRNeasy Mini Kit (Qiagen, Germany). Serum samples were thawed on ice and centrifuged at 12,000g for 5 minutes to remove any cellular debris. For each sample, precisely 200 µL of serum were mixed with 1000 µL Qiazol and 1 µL of a mix of 3 synthetic spike-in controls (Qiagen, Germany). After a 10-minute incubation at room temperature, 200 µL chloroform were added to the lysates followed by cooled centrifugation at 12,000g for 15 minutes at 4°C. Precisely 650 µL of the upper aqueous phase were mixed with 7 µL glycogen (50mg/mL) to enhance precipitation. Samples were transferred to a miRNeasy Mini column, and RNA was precipitated with 750 µL ethanol followed by automated washing with RPE and RWT buffer in a QiaCube liquid handling robot. Finally, total RNA was eluted in 30 µL nuclease free water and stored at -80°C until further analysis.

- Reverse-transcription qPCR (RT-qPCR) analysis

Total RNA samples from serum and CCM were processed identically. Starting from total RNA samples, cDNA was synthesized using the miRCury RT Kit (Qiagen, Germany). Reaction conditions were set according to the manufacturers’ recommendations. In total, 2 µL of total RNA were used per 10 µl reverse transcription (RT) reaction. To monitor RT efficiency and presence of impurities with inhibitory activity, a synthetic RNA spike-in (cel-miR-39-3p) was added to the RT reaction. PCR amplification was performed in a 96-well plate format in a Roche LC480 II instrument (Roche, Germany) using miRCury SYBR® Green mastermix (Qiagen, Germany) with the following settings: 95°C for 2 min, 45 cycles of 95°C for 10 s and 60°C for 60 s, followed by melting curve analysis. To calculate the cycle of quantification values (Cq-values), the second derivative method was used. Cq-values were normalised to the RNA spike-in control level, by subtracting the individual miRNA Cq-value from the RNA Spike-In Cq, thus obtaining delta-Cq (dCq) values which were used for the analysis.

**3. Cell culture**

The epidermoid carcinoma cell line A431 and the oral squamous cell carcinoma cell line SCC4 were purchased from ATCC (Wesel, Germany). The skin squamous carcinoma cell line SCC12 was obtained from Thermo Fisher Scientific (Waltham, MA, USA). Primary human keratinocytes were prepared as described previously (Wagner T et al, [Sci Rep.](https://www.ncbi.nlm.nih.gov/pubmed/?term=PMID%3A+30194332) 2018 Sep 7;8(1):13434). All cells were cultivated in KGM2 medium (Promocell, Heidelberg, Germany) at 37 °C, 5% CO_2_, and at 95% relative humidity. For analysis of released miRNAs, cells were grown in 75cm^2^ culture dishes to 75% confluency and cultivated with 10 ml fresh KGM2 medium for 24 hours.

**4. Statistical analysis**

Group-wise differential expression analysis was performed based on spike-in normalized delta Cq-values (dCqs) under the assumption of normal distribution, which was assessed visually, using two-sided t-tests.

**Figure S1**: Principal component analysis.

Tukey's multiple comparisons testq
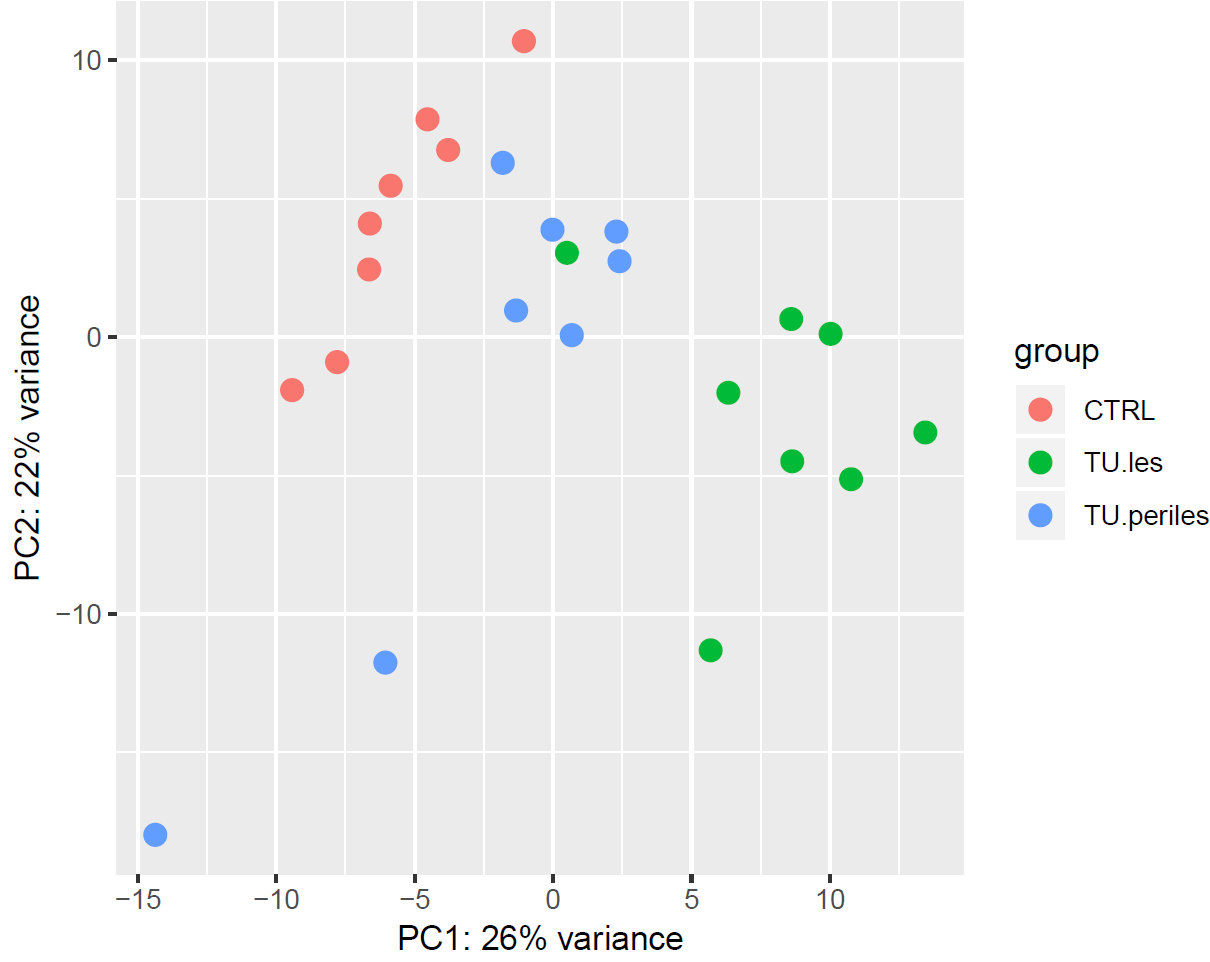


**Figure S1:** The PCA plot shows the first two principal components which explain the variability in the NGSs microRNA data (n=491 microRNAs) using the regularised log count data. The first and second principal component explain 26 and 22 percent of the variance respectively.

CTRL, control (gluteal, UV-unexposed skin); TU.les, tumour lesional tissue; TU.periles, tumour perilesional tissue

**Figure S2**: mir-1246 and miR-1290 expression in cSCC cell lines

**Figure S2:** Mir-1246 and miR-1290 expression in the supernatant of cultured primary keratinocytes (KC) and three different cSCC cell lines (A431, SSC4, SSC12). Supernatants were collected from subconfluent cells and used for differential centrifugation to remove cell debris and larger particles such as apoptotic bodies.
